# Decellularized Aortic Scaffold Alleviates H_2_O_2_-Induced Inflammation and Apoptosis in CD34+ Progenitor Cells While Driving Neovasculogenesis

**DOI:** 10.1155/2020/6782072

**Published:** 2020-02-10

**Authors:** Liping Gao, Anqi Feng, Cui Li, Sebastian Schmull, Hong Sun

**Affiliations:** ^1^Department of Physiology, Xuzhou Medical University, Xuzhou 221004, Jiangsu, China; ^2^National Demonstration Center for Experiment Basic Medical Sciences Education, Xuzhou Medical University, Xuzhou 221004, Jiangsu, China; ^3^Department of Clinical Medicine, Xuzhou Medical University, Xuzhou 221004, Jiangsu, China; ^4^Renji-Med X Clinical Stem Cell Research Center, Ren Ji Hospital, School of Medicine, Shanghai Jiao Tong University, Shanghai 200127, China

## Abstract

Bone marrow-derived stem/progenitor cells have been utilized for cardiac or vascular repair after ischemic injury, but they are subject to apoptosis and immune rejection in the ischemic site. Multiple scaffolds were used as delivery tools to transplant stem/progenitor cells; however, these scaffolds did not show intrinsically antiapoptotic or anti-inflammatory properties. Decellularized aortic scaffolds that facilitate cell delivery and tissue repair were prepared by removing cells of patient-derived aortic tissues. Scanning electron microscopy (SEM) showed cells attached well to the scaffold after culturing for 5 days. Live/dead staining showed most seeded cells survived at day 7 on a decellularized aortic scaffold. Ki67 staining demonstrated that decellularized aortic scaffold promoted proliferation of bone marrow-derived CD34+ progenitor cells. Apoptosis of CD34+ progenitor cells induced by H_2_O_2_ at high concentration was significantly alleviated in the presence of decellularized aortic scaffolds, demonstrating a protective effect against oxidative stress-induced apoptosis. Furthermore, decellularized aortic scaffolds significantly reduced the expression of proinflammatory cytokines (IL-8, GM-CSF, MIP-1*β*, GRO-*α*, Entoxin, and GRO) concurrently with an increase in anti-inflammatory cytokines (IL-2 and TGF-*β*) released from CD34+ progenitor cells when exposed to H_2_O_2_ at low concentration. Finally, neovascularization was observed by H&E and immunohistochemical staining 14 days after the decellularized aortic scaffolds were subcutaneously implanted in nude mice. This preclinical study demonstrates that the use of a decellularized aortic scaffold possessing antiapoptotic and anti-inflammatory properties may represent a promising strategy for cardiovascular repair after ischemic injury.

## 1. Introduction

Ischemic diseases, including myocardial infarction, ischemic stroke, and critical limb ischemia, remain a serious health problem around the world, leading to severe morbidity and mortality [1, 2]. To date, a clear solution to this growing problem is still lacking. Recently, stem cell therapy has emerged as a promising new strategy for the treatment of ischemic diseases. Preclinical studies have shown that bone marrow-derived stem/progenitor cells have favorable effects on cardiac or vascular repair after ischemic injury, presumably through the regeneration of the blood-supplying vasculature [3–5]. Despite these encouraging outcomes, the success of stem cell therapy has been limited due to the low survival rate after transplantation in the ischemic site. It was reported that the majority of transplanted cells undergo apoptosis owing to oxidative stress within the ischemic environment [6–8]. Animal studies revealed a positive correlation between cell survival rate and long-term functional benefit [9], indicating that new strategies to improve the survival of transplanted cells could increase therapeutic benefit. Thus, a platform for stem cell delivery coupled with antiapoptotic capability could be an efficient therapeutic strategy for ischemic diseases.

Scaffolds are commonly used as tools for cell delivery. The cell survival could be enhanced when cells were seeded on scaffold owing to the preservation of cell-cell and cell-extracellular matrix interaction. However, synthetic polymer scaffolds and some natural matrices, such as bovine pericardium, can result in inflammatory reaction [10]. Although inflammation is a necessary component for tissue repair, excessive inflammatory response hinders tissue regeneration. Upregulation of proinflammatory cytokines contribute to pathogenesis after myocardial infarction [11], but the elevation of anti-inflammatory cytokines are associated with improved cell survival in inflammatory and ischemic conditions [12, 13]. Thus, a scaffold with intrinsic anti-inflammatory properties is likely to be therapeutically beneficial to ischemic diseases. Recently, decellularized matrices have been shown to modulate the immune response by exhibiting anti-inflammatory effects. For example, decellularized skeletal muscle scaffolds were found to exert an anti-inflammatory effect as evidenced by reduced IL-2, IFN-*γ*, and raised IL-10 levels in cell-culture supernatants [14]. In a previous study, we prepared decellularized scaffolds from human aortic tissue which was discarded as surgical waste. We found that the decellularized aortic scaffolds possess favorable biocompatibility and induce endothelial differentiation of bone marrow CD34+ progenitor cells [15]. However, the use of decellularized aortic scaffolds to enhance stem cell survival under oxidative conditions and their anti-inflammatory effect is not defined.

In the present study, we utilized decellularized aortic scaffolds as cell delivery vehicles on which the adhesion and proliferation of bone marrow progenitor cells were evaluated. Further, the antiapoptotic and inflammation-modulating properties of decellularized aortic scaffolds were also tested when cells were seeded on scaffolds and exposed to H_2_O_2_ at different concentrations. Our study also analyzed the effect of decellularized aortic scaffolds on vasculogenesis in vivo following subcutaneous implantation in a hind-limb ischemia model. Overall, our results disclosed the potential of decellularized aortic scaffolds as a new therapeutic strategy for ischemic diseases.

## 2. Methods

### 2.1. Preparation of Human Decellularized Aortic ECM

Human aortic tissues were obtained from patients receiving aorta replacement surgery at the Department of Cardiac Surgery of Ren Ji Hospital, Shanghai, China. Our investigations were carried out following the rules of the Declaration of Helsinki. Also, the study including patients' aortic tissues was approved by the Institutional Review Board of Ren Ji Hospital (permit number: 2012027), and patients had signed written informed consent. The patients' information was listed in [Supplementary-material supplementary-material-1]. The decellularized aortic tissues were prepared as described previously [15]. Briefly, patients' aortic tissues were washed in cold saline and cut into 1 cm × 1 cm pieces. Then tissue pieces were cut into 200 *μ*m thick sections using a freezing microtome (CM1950, Leica, Germany). Sections were incubated with 0.5% sodium dodecyl sulfate (Sigma, USA) in deionized water and mildly vortexed at 37°C for 12 h. After rinsing in deionized water, samples were subjected to 200 IU/ml DNase I (Roche, Basel, Switzerland) solution for 4–8 h. Subsequently, the samples were disinfected in 75% ethanol for 2 hours and stored at 4°C until use.

### 2.2. Scanning Electron Microscopy

Scanning electron microscopy (SEM) was utilized to reveal the structural microarchitecture of the decellularized aortic scaffolds and to evaluate cell attachment to the scaffolds. On day 7, the samples were fixed in 2.5% glutaraldehyde solution overnight and then dehydrated by sequential immersion in increasing concentrations of ethanol. Samples were dried in a CO_2_ critical evaporator. Subsequently, the samples were coated with a thin layer of sputtered gold and observed using SEM (Quanta 2000; FEI, USA).

### 2.3. Cell Isolation and Seeding

Bone marrow mononuclear cells were isolated from the bone marrow of mouse tibia by density gradient sedimentation with Mouse Lymphocyte Separation Medium (Dakewe, Shenzhen, China). CD34+ progenitor cells were enriched by magnetic activated cell sorting technique. Hereafter, cells were seeded on aortic scaffolds in 24-well plates as previously described [15]. In brief, 100 *μ*l of CD34+ cell suspension (containing 5 × 10^5^ cells) was added dropwise on the surface of each scaffold, allowing cells to distribute throughout the surface of aortic scaffold or fibronectin-coated culture plates. Cell-seeded scaffolds were kept at 37°C in a CO_2_ incubator for 2 h in order to enable the cells to attach to the scaffolds. Next, 900 *μ*l of culture medium was added in each well, and incubation was resumed. CD34+ progenitor cells in all groups were cultured in Iscove's Modified Dulbecco's Medium containing 10% (v/v) fetal bovine serum (Biowest, MO, USA).

### 2.4. Live/Dead Staining

On day 3 or day 7, the survival of cells on the decellularized aortic scaffolds was evaluated using live/dead cell viability assay (Life Technologies, MA, USA), respectively. After removing the medium, cells were washed once with PBS and then incubated with 200 *μ*L of live/dead reagent containing 4 *μ*M Calcein AM and 8 *μ*M PI at 37°C for 20 min. Subsequently, the cells were washed with PBS and examined through a fluorescence microscope (Nikon, Tokyo, Japan). Live cells showed up green while dead cells appeared red under fluorescence microscopy.

### 2.5. Cell Proliferation

The proliferation of CD34+ cells on scaffolds was detected at day 7. Cells were fixed in 4% paraformaldehyde for 15 min, permeabilized in 0.5% Triton X-100, and blocked with 5% normal donkey serum for 1 h. Hereafter, samples were incubated with primary antibody mouse anti-Ki67 (BD Biosciences, CA, USA) overnight at 4°C. The secondary antibody donkey antimouse Alexa488 (Life Technologies, USA) was added to the samples and incubated for 1 h. DAPI (Sigma, St. Louis, USA) was used to counterstain the cell nuclei.

### 2.6. Cell Apoptosis Detection

Apoptosis of cultured CD34+ progenitor cells was induced via exposure to a high level of H_2_O_2_ [16]. After being cultured in Iscove's Modified Dulbecco's Medium containing 50 *μ*mol/L or 100 *μ*mol/L H_2_O_2_ (323381, Sigma-Aldrich, St. Louis, USA) for 24 hours, CD34+ progenitor cells of the two groups (CD34+ cells on fibronectin and CD34+ cells on aortic scaffold) were washed with ice-cold PBS and digested in 0.25% trypsin solution. The collected cell suspension was centrifuged at 1500 rpm for 5 min, then the supernatant was removed, and cell mass was resuspended with binding buffer. After addition of 5 *μ*l Annexin V-FITC *T* and 10 *μ*l PI successively, cells were incubated for 15 minutes at room temperature in the dark. The stained samples were immediately analyzed using a flow cytometer (BD Accuri C6, BD biosciences, NJ, USA) after being diluted with 400 *μ*L of binding buffer for each sample. A total of 10^5^ cells for each sample were counted, and the cell apoptosis rate was calculated.

### 2.7. Cytokine Detection

The cells in the CD34 group (CD34+ cells on fibronectin) and the CD34+ scaffold group (CD34+ cells seeded on the aortic scaffold) were transferred to Iscove's Modified Dulbecco's Medium containing 0.5% FBS and 25 *μ*mol/L H_2_O_2_ on day 4 and then cultured for 48 h. The medium was collected and concentrated 5 times by centrifugation at 4°C for 25 min at 3000 g using Amicon Ultra 4 centrifugal filter tubes with 3 kDa nominal molecular weight limit membranes (Millipore, MA, USA). Relative levels of 120 cytokines were determined using a Sandwich immunoassay array according to the manufacturer's instructions (Mouse Cytokine Antibody Array C Series 1000; Raybiotech, Inc., USA). The identified cytokines are listed in [Supplementary-material supplementary-material-1]. Chemiluminescence and signal intensities were detected using an Image Quant LAS4000 Scanner (GE Healthcare Life Sciences, PA, USA).

### 2.8. Hind-Limb Ischemia Murine Model

The present animal experiments were approved by the Institutional Animal Care and Use Committee at Ren Ji Hospital, Shanghai, China (Approval number: 2014-13k-012). To detect vasculogenesis in vivo, the cell-aortic scaffold constructs were implanted into nude mice. Matrigel assay, the preferred in vitro angiogenesis model [17], is considered as a comparison. All 6-week-old male nude mice (Slrc laboratory animal, Shanghai, China) were anesthetized with isoflurane. The femoral artery and its branches were ligated with 6-0 silk, and then the femoral artery was excised from its proximal origin to the distal point where it bifurcates into the saphenous and popliteal arteries [18]. Immediately after the ischemic surgery, the scaffolds or Matrigel containing CD34+ cells were implanted at a subcutaneous site of the medial thigh of each mouse. There were three groups, including the control group (only decellularized aortic scaffold were implanted), the CD34+ Matrigel group (CD34+ cells and Matrigel were implanted), and the CD34+ scaffold group (CD34+ cells and decellularized aortic scaffold were implanted). After the placement of implants, skin incisions were closed with 4-0 nylon suture. After 14 days mice were euthanized and the ischemic limb tissues were retrieved.

### 2.9. Histological and Immunohistochemical Staining

To investigate the potential of inductive neovasculogenesis in vivo, histological and immunohistochemical staining of the scaffold was performed as previously described [15]. Briefly, the samples from all three groups were fixed in 4% paraformaldehyde, embedded in paraffin, and then cut into 5 *μ*m sections. Hereafter, the slides were placed in citrate antigen retrieval buffer and heated to 95°C for 20 min. After cooling to room temperature, sections were treated with 3% hydrogen peroxide solution for 20 min and then placed in a humidity chamber to incubate for 30 min with 10% normal goat serum. Subsequently, sections were incubated with anti-Von Willebrand Factor antibody (VWF, Abcam, Cambridge, UK) or antialpha smooth muscle actin (*α*-SMA, Abcam, Cambridge, UK) at 4°C overnight. After sections were washed with PBS, they were subjected to a biotinylated secondary antibody for 30 min. Hereafter, sections were rinsed in PBS, and diaminobenzidine solution was applied. After staining, slides were dehydrated and cover-slipped and then imaged using a microscope.

### 2.10. Statistical Analysis

Data were collected and analyzed using GraphPad prism 5.0 software (GraphPad software, Inc. CA, USA). All data were presented as mean ± standard error. Three replicates for each experiment (cell proliferation, H_2_O_2_-induced apoptosis, and protein expression of inflammatory cytokines) were performed. A student's *t* test was employed to examine the difference of quantitative data between the two groups. *P* < 0.05 was set as a threshold to indicate statistical significance.

## 3. Results

### 3.1. Decellularized Aortic Scaffold Enhanced Cell Adhesion and Cell Survival

**Figure 1:**
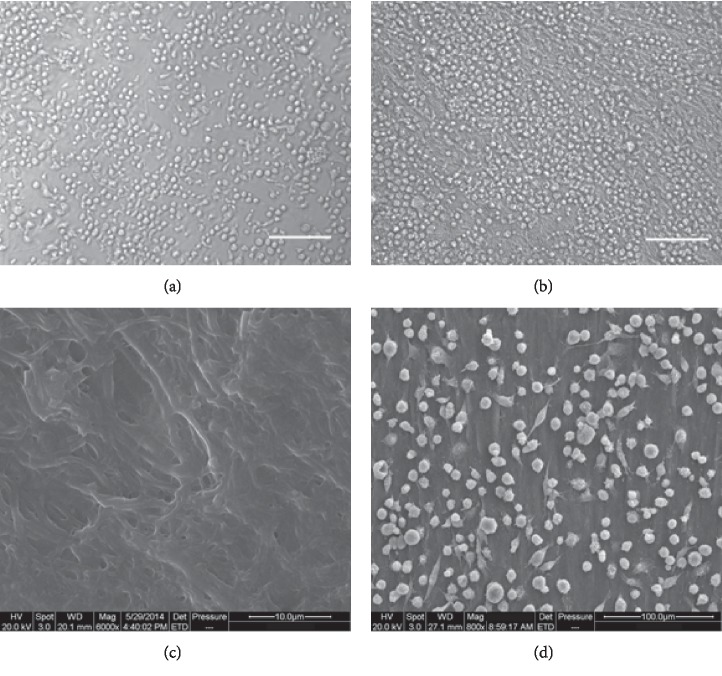
Morphological feature and adhesion of CD34+ progenitor cells on the decellularized aortic scaffold. (a and b) Representative images of CD34+ progenitor cells on fibronectin (a) and decellularized aortic scaffold (b) on day 5 under an optical microscope. Scale bar: 100 *μ*m. (c) SEM image showing the microstructure of decellularized aortic scaffold at high magnifications. (d) SEM showing the adhesion of CD34+ progenitor cells on the decellularized aortic scaffold at day 5 after seeding.

**Figure 2:**
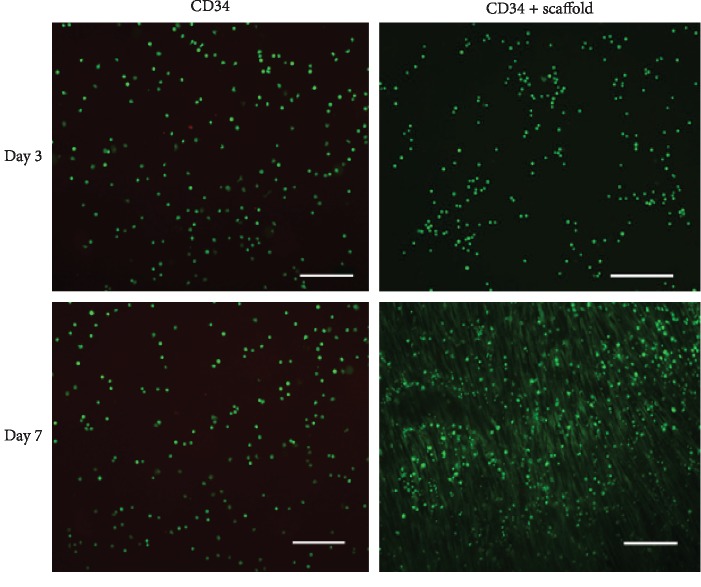
Survival of CD34+ progenitor cells on the decellularized aortic scaffold. CD34+ progenitor cells on fibronectin or decellularized aortic scaffold were stained with Calcein AM (green) or PI (red) at day 3 or day 7 after seeding. Scale bar: 100 *μ*m.

We removed all cells of human aortic tissue using detergent and enzyme and prepared a decellularized aortic scaffold. The microscopic morphology of decellularized aortic scaffold with or without seeding of CD34+ progenitor cells was displayed in Figures 1(a) and 1(b). SEM showed that a decellularized aortic scaffold possessed a three-dimensional porous structure at high magnification. There were a large number of cells present on the surface of the decellularized aortic scaffold and these cells attached well to the scaffold after culturing for 5 days. Live/dead cell staining demonstrated that the vast majority of cells survived on the decellularized aortic scaffold at day 5 or day 7, as shown in Figure 2.

### 3.2. Decellularized Aortic Scaffold Promoted Proliferation of CD34+ Progenitor Cells

**Figure 3:**
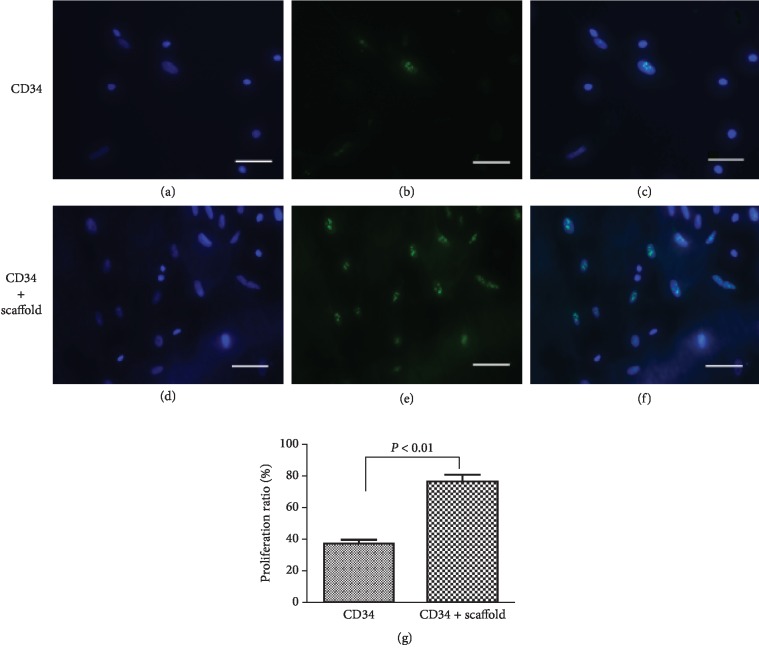
The proliferation of CD34+ progenitor cells on decellularized aortic scaffolds. Cell proliferation was assessed by Ki67 fluorescence staining for CD34+ on scaffolds. Representative images showing proliferation of CD34+ progenitor cells on fibronectin (a–c) and on decellularized aortic scaffolds (d–f). Cell nuclei are indicated in blue, and Ki67-positive cells in green. (g) Proliferation ratios (percentages of Ki67-positive CD34+ cells) on aortic scaffold or fibronectin were calculated at day 10 after seeding from 3 different experiments. *P* < 0.01. Scale bar: 100 *μ*m.

Immunofluorescence assay was performed on day 10 to evaluate the proliferation rate of CD34+ cells on scaffold or fibronectin, respectively. Ki67, a nuclear protein used as a marker for proliferation, was detected in CD34+ progenitor cells in both groups. It was found that Ki67 expression in the scaffold group was much higher than that in the control group (*P* < 0.01, Figure 3), which suggested that decellularized aortic scaffold promoted proliferation of CD34+ progenitor cells. These data from cell adhesion, survival, and proliferation demonstrate the feasibility of decellularized aortic scaffold being used as cell carriers for bone marrow stem/progenitor cells in cell transplantation therapies.

### 3.3. Decellularized Aortic Scaffold Decreased Apoptosis of CD34+ Progenitor Cells When Exposed to High Level of H_2_O_2_

**Figure 4:**
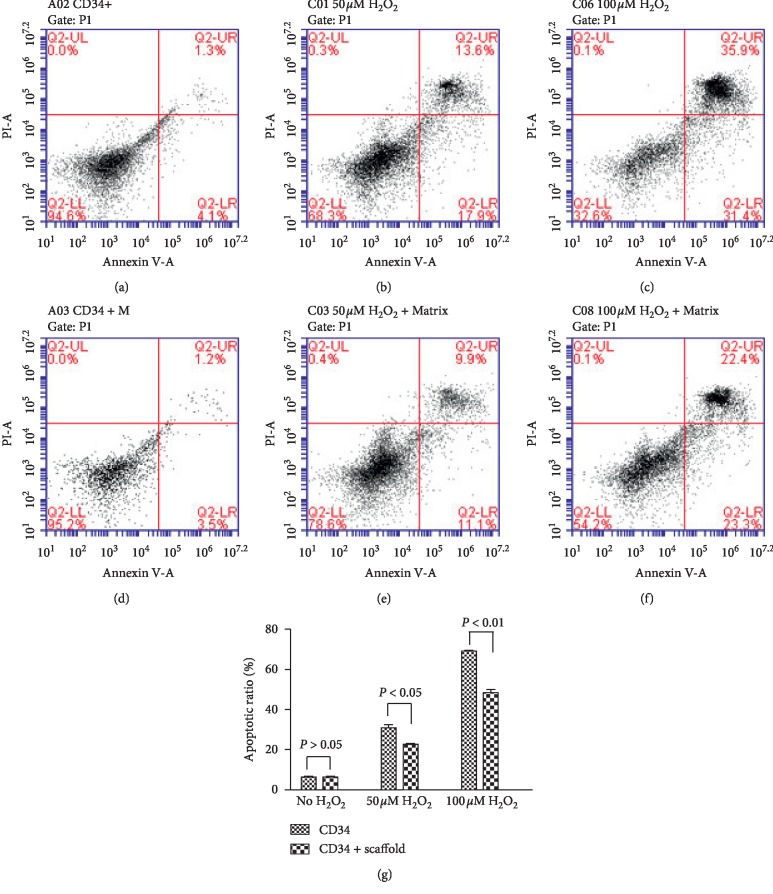
Decellularized aortic scaffold inhibited H_2_O_2_-induced apoptosis in vitro. Apoptosis was detected by FACS in CD34+ progenitor cells treated with 50 *μ*mol/L or 100 *μ*mol/L H_2_O_2_ in the absence or presence of decellularized aortic scaffold. (a) CD34+ cells only. (b) CD34+ cells exposed to 50 *μ*mol/L H_2_O_2_. (c) CD34+ cells exposed to 100 *μ*mol/L H_2_O_2_. (d) CD34+ cells in the presence of a decellularized aortic scaffold. (e) CD34+ cells exposed to 50 *μ*mol/L H_2_O_2_ in the presence of a decellularized aortic scaffold. (f) CD34+ cells exposed to 100 *μ*mol/L H_2_O_2_ in the presence of a decellularized aortic scaffold. (g) Apoptotic ratios (percentages of apoptotic CD34+ cells) were calculated in both groups when treated with no H_2_O_2_, 50 *μ*mol/L H_2_O_2_, or 100 *μ*mol/L H_2_O_2_. Compared with CD34 group, *P* > 0.05, *P* < 0.05, *P* < 0.01, respectively.

The effect of decellularized aortic scaffold on H_2_O_2_-induced apoptosis was analyzed using flow cytometry. Under basal conditions, the apoptotic rate of CD34+ cells was very low and there was no difference for apoptotic rate with or without aortic scaffold (*P* > 0.05), Figures 4(a) and 4(d). However, the apoptotic rate of CD34+ cells was significantly elevated when cells were exposed to 50 or 100 *μ*mol/L of H_2_O_2_ for 24 hours (Figures 4(b), 4(c), 4(e), and 4(f)). Particularly, the apoptotic rate of CD34+ cells on the aortic scaffold was significantly declined compared with that of CD34+ cells without scaffold after incubation with H_2_O_2_, as shown in Figure 4(g), which indicated an inhibitory function of the aortic scaffold to oxidative stress-induced apoptosis.

### 3.4. Effects of Decellularized Aortic Scaffold on Expression of Inflammatory Cytokines Secreted by CD34+ Progenitor Cells When Exposed to Low Concentration of H_2_O_2_

**Figure 5:**
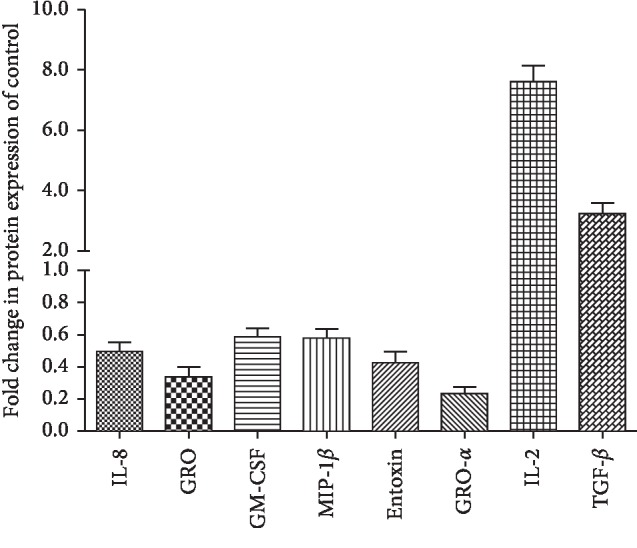
Relative protein expression levels of inflammatory cytokines secreted by CD34+ progenitor cells in the presence of a decellularized aortic scaffold. Protein expression was evaluated using a protein chip analysis. In the absence of a decellularized aortic scaffold, the protein levels of inflammatory cytokines released from CD34+ progenitor cells exposed to 25 *μ*mol/L H_2_O_2_ were used as control. Data are representative of 3 biological replicates.

The inflammatory reaction is an essential step in response to ischemic injury. It was well known that stem cells exert therapeutic effects mainly by secreting various cytokines including anti-inflammatory factors [19]. To investigate the effect of a decellularized aortic scaffold on the secretion of inflammatory cytokines, CD34+ progenitor cells were exposed to H_2_O_2_ at low concentration in the presence and absence of a decellularized aortic scaffold. We found that CD34+ progenitor cells released inflammatory cytokines after they were cultured in a culture medium containing 25 *μ*mol/L H_2_O_2_ for 48 hours (data not shown). Among 120 cytokines, a significant decrease in expression of key proinflammatory cytokines, including IL-8, GM-CSF, MIP-1*β*, GRO-*α*, Entoxin, and GRO, were observed in supernatant of the CD34+ cell-scaffold group as compared to the CD34+ cell group. Meanwhile, the secretion of anti-inflammatory cytokines, IL-2 and TGF-*β*, was significantly increased by CD34+ progenitor cells on aortic scaffold compared with CD34+ cell only, as indicated in Figure 5. These results indicated that the human aortic scaffold has intrinsic anti-inflammation property as evidenced by the downregulation of proinflammatory cytokines and the upregulation of anti-inflammatory cytokines, which contributes to enhanced survival of stem cells.

### 3.5. Microvessel Formation inside Aortic Scaffold after Transplantation into Nude Mice

**Figure 6:**
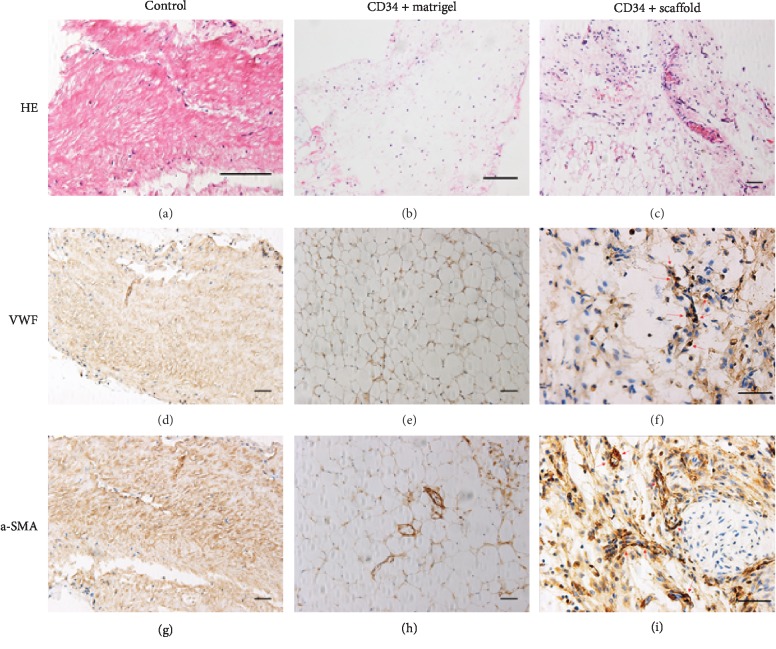
CD34+ progenitor cell transplantation using a decellularized aortic scaffold induces vascularization in a hind-limb ischemia model of nude mice. CD34+ progenitor cells were mixed with Matrigel (CD34+ Matrigel group) or seeded onto the decellularized aortic scaffold and subcutaneously implanted into nude mice. The decellularized aortic scaffold without cell seeding was used as the control group. After 2 weeks of implantation, the ischemic limb tissues were retrieved for histological analysis (a–c). H&E staining indicated the presence of many microvessels with functionality in the aortic construct (many red blood cells can be seen in these microvessels). The in vivo differentiation of CD34+ cells into ECs and SMCs was detected by immunohistochemical staining for VWF and *α*-SMA (d–i). VWF-positive or *α*-SMA-positive cells in the CD34+ scaffold group were indicated by red arrows. Scale bar: 100 *μ*m.

After 2 weeks of subcutaneous implantation in nude mice, the ischemic limb tissues were harvested and subjected to histological and immunohistochemical staining. H&E staining showed that microvessel tissues formed in the CD34+ scaffold group but did not in the Matrigel group and the control group (Figures 6(a)–6(c)). A large number of red blood cells were observed inside the vessel tissues in the CD34+ scaffold group, indicating that neomicrovessels had been generated in the ischemic tissues after subcutaneous implantation of CD34+ cell-scaffold construct for 2 weeks. Positive staining for VWF and *α*-SMA was observed in many cells inside the CD34+ scaffold group, which further presented evidence of aortic scaffold-induced neovascularization (Figures 6(d)–6(i)).

## 4. Discussion

Decellularized matrix scaffolds have been widely utilized to repair ischemic tissues and promote tissue regeneration [20–22]. The prevailing advantage of decellularized matrix scaffolds is their similar properties with the native extracellular matrix, thus, providing a proper microenvironment for cell survival, retention, and engraftment of transplanted stem cells [23]. In this study, decellularized scaffolds were prepared from human aortic tissues. In vitro experiments indicated that the decellularized aortic scaffolds have good biocompatibility and facilitate cell adhesion and proliferation. More importantly, decellularized aortic scaffolds were shown to have intrinsic anti-inflammatory and antiapoptotic properties. Additionally, the decellularized aortic scaffolds promoted the formation of functional microvessels inside the ischemic tissues when they were subcutaneously implanted into nude mice. These outcomes demonstrated that decellularized aortic scaffold may be used as a favorable material for ischemic injury repair and tissue regeneration.

Inflammation will drastically alter the immune environment within the implant and can adversely affect the survival and function of transplanted cells. In ischemia-related pathologies, most of the cytokines serve as biological markers of inflammation. Some inflammatory cytokines, including IL-1, IL-4, IL-6, IL-8, IL-18, and TGF-*β*1, are involved in the development of ischemic heart disease, myocardial infarction, and heart failure, thus, serving as potential targets for the development of some anti-ischemic therapies [24]. It was reported that the decellularized matrix has immunomodulatory properties and the ability to influence the host response in vivo [25]. Thus, we performed a protein chip assay to detect the expression of proinflammatory cytokines and anti-inflammatory cytokines when a cell or cell-scaffold construct was exposed to H_2_O_2_. Our chip results showed that aortic scaffold enhanced the expression of anti-inflammatory cytokines such as IL-2 and TGF-*β*. It was well established that IL-2 can limit inflammation and prevent the uncontrolled expansion of immune responses by inhibiting IL-17-dependent inflammatory processes as well as interfering with IL-6-dependent signaling events [26]. Administration of low-dose IL-2 can diminish inflammation and ameliorate disease in patients suffering from chronic graft-versus-host disease [27]. TGF-*β* exerts the anti-inflammatory function mainly by inhibiting the migration and proliferation of neutrophils and macrophages and suppressing T cell maturation. A recent investigation demonstrated that TGF-*β*1 embedded in scaffolds or nanoparticles reduced inflammation and enhanced function of transplanted cells in cell-based therapies [28, 29]. Meanwhile, our experiment also found that decellularized aortic scaffold inhibited the secretion of proinflammatory cytokines, including IL-8, GM-CSF [30], MIP-1*β*, GRO-*α*, GRO [31], and Entoxin [32]. These results indicate the protective property of decellularized aortic scaffold in balancing the proinflammatory and anti-inflammatory cytokines in favor of an anti-inflammatory microenvironment. Therefore, the decellularized aortic scaffold shows intrinsic anti-inflammatory property and may alter the inflammatory response in the site of ischemic tissue and subsequently promote tissue regeneration.

Previous studies found that about one-third of grafted cells inside the injured heart is positive for TUNEL staining one day after transplantation [33], which suggested that these transplanted cells mainly experienced apoptosis in the hostile environment. Amsalem et al. also reported that most labeled cell grafts are lost within 4 weeks of transplantation due to their inability to withstand oxidative stress-induced apoptosis in the ischemic myocardium [34]. Therefore, a strategy with an antiapoptotic feature is promising to enhance cell survival and improve therapeutic efficacy in ischemic diseases. To mimic the oxidative stress microenvironment in ischemic tissue, cells were exposed to H_2_O_2_ at different concentrations and cultured in the presence and absence of aortic scaffolds. In conditions without H_2_O_2_, cell apoptosis rates in the control group and the scaffold group by FACS were about 4.5% and 4.9%, respectively. However, live and dead staining results showed that almost no dead cell was observed when cultured for 7 days. This discrepancy may be due to the different manipulation steps in the two experiments. Before FACS detection, cells need to be collected and centrifugated, which may damage cells and lead to cell death. Yet, for live and dead staining, cells did not experience these steps. Cell apoptosis rate in the control group reached 48.5 ± 5.5% and 61.4 ± 8.6% in medium containing 50 or 100 *μ*mol/L H_2_O_2_ and markedly decreased to 36.5 ± 5.5% and 50.4 ± 5.5% in the scaffold group, respectively. These observations indicated that a decellularized aortic scaffold could greatly reduce the apoptosis rate and enhance the survival of CD34+ progenitor cells. Considering that cellular apoptosis may be triggered by the disruption of cell-cell and cell-extracellular matrix interactions [7, 23], we speculate that aortic scaffold provides a substrate which contains some biological cues for stem cell adhesion and growth, thus, protecting cells from noxious insults like ischemia and reducing cell death due to anoikis. However, the exact mechanisms are still unclear and deserve further investigation.

Transplanted cells depend on oxygen and nutrients provided by microvessels. Without vascularization, transplanted cells will undergo apoptosis [35]. Therefore, tissue neovascularization appears extremely important to the long-term survival of the implants and seeded cells. To evaluate the capacity of inductive neovascularization, we implanted the aortic scaffold into a hind-limb ischemia model of nude mice. At 14 days after implantation, H&E and immunohistochemical staining demonstrated the formation of functional neomicrovessels inside the ischemic tissues. In addition, a visually higher density of neovascularization was observed in the cell + aortic scaffold group when compared to the Matrigel group, which indicated decellularized aortic scaffolds have good capability of inducing neovascularization. However, neovascularization was not found in the control group (only scaffold implanted) in Figure 6, suggesting the seeded CD34+ progenitor cells also play an important role in the neovascular formation. Although the seeded cells on implants may be lost after implantation, they could promote tissue regeneration and neovascularization via secreting multiple cytokines [36, 37]. Based on these findings, we consider that both the seeded CD34+ cells and aortic scaffold contribute to the neovascular formation in the hind-limb ischemia model of nude mice.

## 5. Conclusions

Our study revealed that decellularized aortic scaffolds could promote cell attachment and cell survival, alleviate inflammatory reaction, inhibit the apoptosis of bone marrow-derived progenitor cells, and promote neovascularization *in vivo*. Thus, the usage of decellularized aortic scaffolds may be an efficient strategy for stem cell therapies of ischemic diseases.

## Data Availability

The data used to support the findings of this study are available from the corresponding author upon request.

## Supplementary Materials

Supplementary Materials Table S1: information of patients who provided aortic tissues. Table S2: cytokine secretion profile of CD34+ EPC exposed to H_2_O_2_ for 48 hours.
