# Supplementary material for: Decellularized Aortic Scaffold Alleviates H2O2-Induced Inflammation and Apoptosis in CD34+ Progenitor Cells While Driving Neovasculogenesis
Source: Biomed Res Int. 2020 Feb 10;2020:6782072. doi: 10.1155/2020/6782072 (PMC7035506; doi:10.1155/2020/6782072)
Supplement: Supplementary Materials — Table S1: information of patients who provided aortic tissues. Table S2: cytokine secretion profile of CD34+ EPC exposed to H2O2 for 48 hours. [file 6782072.f1.pdf]

Supplementary Table S1. The information of patients who provided aortic tissues.

Patient number	Sex	Age	Diagnosis	Sample
1	male	60	coronary disease	Aortic wall
2	male	50	coronary disease; inferior wall myocardial infarction	Aortic wall
3	male	64	coronary disease	Aortic wall
4	female	59	ascend aortic aneurysm; aortic regurgitation; mitral regurgitation; Bentall's operation	Ascending aortic wall
5	male	60	coronary disease; percutaneous coronary intervention (PCI) and Off-pump coronary artery bypass (OPCAB)	Aortic wall
6	male	44	aortic dissection（Debakey Ⅰ）	Aortic wall
7	female	27	ascend aortic aneurysm; aortic insufficiency; Marfan syndrome	Aortic wall
8	female	56	aortic dissection（Debakey Ⅰ）	Aortic wall

Supplementary Table S2. Cytokine secretion profile of CD34+ EPC exposed to H_2O_2 for 48 hours.

The presence and relative levels of a total of 120 cytokines in Control group and aortic scaffold group were screened using a commercially available cytokine antibody array C series 1000 (Ray Biotech, USA). The expression of each cytokine was measured by chemiluminescence intensity by Image Quant LAS4000 Scanner (GE Healthcare Corporate). The fold-increase over Control group was expressed in column 3 (Scaffold group / Control group), values were the average of three experiments.

Cytokines	Control group	Scaffold group	Fold-increase	Cytokines	Control group	Scaffold group	Fold-increase
Angiogenin	237	194	0.82	Acrp30	1595	1616	1.01
BDNF	1009	956	0.95	AgRP	767	744	0.97
BLC	350	312	0.89	Angiopoietin-2	1363	1351	0.99
BMP-4	871	709	0.81	Amphiregulin	364	355	0.98
BMP-6	351	290	0.83	axl	228	195	0.85
CK beta 8-1	1015	868	0.86	bFGF	1790	1543	0.86
CNTF	753	633	0.84	Beta-NGF	209	171	0.82
EGF	457	339	0.74	BTC	567	496	0.87
Eotaxin	465	242	0.52	CCL28	218	122	0.56
Eotaxin-2	544	536	0.98	CTACK	3594	3981	1.11
Eotaxin-3	683	751	1.10	dtk	526	644	1.23
FGF-6	837	1011	1.21	EGF-R	147	168	1.15
FGF-7	417	479	1.15	ENA-78	539	520	0.96
Flt-3 Ligand	498	506	1.02	Fas/TNFRSF6	594	687	1.16
Fractalkine	654	658	1.01	FGF-4	491	446	0.91
GCP-2	565	530	0.94	FGF-9	2237	2207	0.99
GDNF	602	586	0.97	G-CSF	786	820	1.04
GM-CSF	118	73	0.62	GITR ligand	455	429	0.94
I-309	278	240	0.86	GITR	324	314	0.97
IFN-gamma	266	236	0.89	GRO	5143	1974	0.38
IGFBP-1	315	268	0.85	GRO-alpha	2679	750	0.28
IGFBP-2	841	715	0.85	HCC-4	355	376	1.06
IGFBP-4	587	417	0.71	HGF	123	124	1.00
IGF-I	414	305	0.74	ICAM-1	777	674	0.87
IL-10	95	93	0.98	ICAM-3	293	320	1.09
IL-13	125	139	1.11	IGF-BP-3	1376	1532	1.11
IL-15	488	503	1.03	IGF-BP-6	1943	2053	1.06

IL-16	506	540	1.07	IGF-I SR	548	635	1.16
IL-1alpha	357	346	0.97	IL-1 R4/ST2	2385	2822	1.18
IL-1beta	594	672	1.13	IL-1 RI	287	354	1.23
IL-1ra	1266	1391	1.10	IL11	309	314	1.02
IL-2	66	459	6.91	IL12-p40	1429	1362	0.95
IL-3	541	530	0.98	IL12-p70	505	417	0.83
IL-4	349	389	1.12	IL17	206	229	1.11
IL-5	118	123	1.04	IL-2 Ra	333	252	0.76
IL-6	71	84	1.19	IL-6 R	446	315	0.71
IL-7	94	107	1.15	IL-8	8395	3847	0.46
Leptin	551	456	0.83	I-TAC	928	1041	1.12
LIGHT	608	491	0.81	Lymphotactin	1365	1440	1.05
MCP-1	1204	1169	0.97	MIF	876	867	0.99
MCP-2	467	488	1.05	MIP-1-alpha	5604	4986	0.89
MCP-3	261	307	1.17	MIP-1-beta	12144	7650	0.63
MCP-4	315	329	1.05	MIP-3-beta	1269	1434	1.13
M-CSF	666	726	1.09	MSP-a	224	227	1.01
MDC	661	814	1.23	NT-4	301	294	0.98
MIG	146	172	1.18	Osteoprotegerin	574	664	1.16
MIP-1-delta	221	242	1.09	Oncostatin M	3058	3016	0.99
MIP-3-alpha	243	256	1.06	PlGF	283	249	0.88
NAP-2	718	766	1.07	sgp130	405	405	1.00
NT-3	1216	1104	0.91	sTNF RII	1083	905	0.84
PARC	886	655	0.74	sTNF-RI	694	512	0.74
PDGF-BB	735	637	0.87	TECK	658	696	1.06
RANTES	763	718	0.94	TIMP-1	3416	3173	0.93
SCF	529	553	1.05	TIMP-2	882	520	0.59
SDF-1	414	458	1.10	TPO	1315	1301	0.99
TARC	578	687	1.19	TRAIL-R3	462	511	1.11
TGF-beta 1	284	309	1.09	TRAIL-R4	340	376	1.11
TGF-beta	406	1502	3.7	uPAR	664	562	0.85
TNF-alpha	152	187	1.23	VEGF	2077	1950	0.94
TNF-beta	312	375	1.20	VEGF-D	1278	1374	1.08
